# *HORMAD2* methylation‐mediated epigenetic regulation of gene expression in thyroid cancer

**DOI:** 10.1111/jcmm.13680

**Published:** 2018-07-24

**Authors:** Qiuyu Lin, Sen Hou, Feng Guan, Chenghe Lin

**Affiliations:** ^1^ Department of Nuclear Medicine The First Hospital of Jilin University Changchun China

**Keywords:** 5‐aza‐2′‐deoxycytidine, DNA methylation, *HORMAD2*, thyroid cancer

## Abstract

This study is aimed to investigate the methylation level of candidate genes and its impact on thyroid carcinoma (THCA) development. Infinium Human Methylation 450 BeadChip Arrays by Illumina (Illumina HM450K) was the most popular CpG microarray platform widely used in biological and medical research. The methylation level of differentially expressed genes and their corresponding CpG sites were analysed by R programme. The expression of *HORMAD2* was evaluated by qRT‐PCR and Western blot, while the methylation level was examined via methylation‐specific PCR. Cell viability, metastasis, cell cycle and apoptosis were detected by MTT assay, transwell and wound healing assay and flow cytometry, respectively, after treatment with 5‐aza‐2′‐deoxycytidine (5‐Aza). Tumour formation assay was used to analyse thyroid tumour growth in nude mice in vivo. The methylation levels of all 116 differentially expressed genes were analysed. *HORMAD2* was significantly hypermethylated and its mRNA expression was inhibited in THCA cells. After treatment with 5‐Aza, *HORMAD2* expression was up‐regulated in THCA cells and its overexpression can suppress thyroid cancer cell viability, mobility and invasiveness remarkably. Up‐regulation of *HORMAD2* in THCA cells could prolong G0/G1 phase and shorten S phase to impede cell mitosis as well as promote thyroid cancer cells apoptosis. Furthermore, tumour formation assay showed that increased *HORMAD2* level impeded tumour growth in vivo. Hypermethylation of *HORMAD2* could induce THCA progression, while hypomethylation of *HORMAD2* retard cell growth and mobility and facilitate apoptosis through increasing its mRNA expression.

## 1 INTRODUCTION

Thyroid cancer (THCA) is the most pervasive malignant neoplasm in the endocrine system worldwide, accounting for 90% of all endocrine tumours.^1^ The majority of thyroid carcinomas are derived from follicular cells having a spectrum of differentiation from well‐differentiated carcinomas follicular thyroid carcinoma (FTC) and papillary thyroid carcinoma (PTC) to the most invasive and less frequently diagnosed malignancy poorly differentiated thyroid carcinoma (PDTC) and anaplastic thyroid carcinoma (ATC).^2^ To date, the incidence of thyroid carcinoma is still on the rise for uncertain reasons. Some researchers have supposed that it might be associated with epigenetic events.^3^, ^4^, ^5^ As THCA recurrence is a common event in thyroid carcinoma patients, especially in the early stage.^6^, ^7^ Therefore, it is imperative to figure out the pathogenesis of THCA for developing effective diagnostic and therapeutic strategies.

DNA methylation is an epigenetic process linked to the regulation of several biological events, including transcriptional regulation of gene expression, X chromosome inactivation, genomic “imprinting,” silencing endogenous retroviruses and so forth.^8^ The alterations in DNA methylation status in cancer cells are characterized by promoter CpG island hypermethylation and diffuse genomic hypomethylation, which has emerged as an important mechanism involved in cancer progression. Hypermethylation of promoter regions of crucial genes can repress relevant gene expression and influence biological functions, thereby facilitating tumorigenesis.^9^ For instance, Wehbe et al^10^ disclosed that interleukin‐6 promoted cholangiocarcinoma cell growth by aberrant promoter methylation and gene expression. Watson et al^11^ reported that increased DNA methylation in the *HoxA5* promoter region contributed to tumour growth via down‐regulation of *HoxA5* expression. Lal et al demonstrated that *RIZ1* was epigenetically inactivated by promoter hypermethylation in thyroid carcinoma and identified as a potential novel therapeutic target.^12^ Thus, aberrant promoter methylation may be an effective way to detect candidate targets for THCA treatment.

In recent years, the deeper insights into THCA and the rapid development of molecular detection technology have allowed analyses of THCA at the molecular level. Genome‐wide expression analysis has been successfully adopted to identify molecular signatures, improving the diagnosis and prognosis of several types of tumours.^13^ Considerable researches have explored the mechanism of certain genes in multiple types of cancers via genome‐wide microarray analysis of gene expression profiling. For instance, Hou et al^14^ uncovered an epigenetic mechanism that *BRAF* could promote PTC carcinogenesis through modulating the methylation and the expression of multiple crucial genes. Nikolova et al^15^ revealed the significant role of *RGS4* in thyroid carcinogenesis and identified molecular targets for THCA treatment through genome‐wide gene expression profiles. HORMA‐domain containing 2 (*HORMAD2*) was a conserved meiotic chromosomal protein‐coding gene in many organisms, including yeast, plants, nematodes and mammals.^16^ Nonetheless, few studies focused on the molecular mechanism of *HORMAD2* from the perspective of whole genome DNA methylation and epigenetic regulation of gene expression.

The purpose of present research is to probe into the methylation level of candidate genes and the effects on THCA progression. We first identified differentially expressed genes (DEGs) and relevant CpG sites via bioinformatics analysis via R programme. Then, DNA methylation level in tumour and normal tissues was detected by methylation‐specific PCR (MSP). Subsequently, we observed significantly hypermethylated *HORMAD2*, of which the expression was examined by qRT‐PCR and Western blot. Furthermore, 5′‐aza‐2′‐deoxycytidine was utilized to investigate the impact on the methylation level of *HORMAD2* and biological functions in THCA.

## 2 MATERIALS AND METHODS

### 2.1 DNA methylation profiling

A portion of these DNA samples were isolated from thyroid cancer tissues and adjacent normal tissues in 507 patients (136 male and 371 female). The DNA methylation statuses of 108 DNA samples in total that were hybridized in the Infinium Human Methylation 450 BeadChip were evaluated, and then we integrated the data sets and removed probes that did not exist in the data sets. Furthermore, approximately 200 000 CpGs were abandoned according to SNPs, repeats and multiple mapping sites. The final set incorporated above 180 000 unique probes.

### 2.2 CHARM (comprehensive high‐throughput arrays for relative methylation)

Comprehensive high‐throughput arrays for relative methylation was applied to McrBC analysis, the array design and computational algorithms are fractionation method‐independent and make this a simple, general, relatively inexpensive tool suitable for genome‐wide analysis, and in which individual samples can be assayed reliably at very high density, allowing locus‐level genome‐wide epigenetic discrimination of individuals, not just groups of samples. The process of establishing the array was as follows: (*i*) all the CpGs in the genome were identified and any region of 300 bp with no CpGs was removed. (*ii*) Probes with multiple matches including fuzzy matches as defined by NimbleGen were discarded to the genome. (*iii*) Any region of 300 bp between sequential probes was divided into two new regions, while any region with fewer than 15 probes was removed. (*iv*) Regions were tiled using 50‐mers 35 bp apart.

### 2.3 Data processing and bioinformatics analysis

The β values, or the percentage of CpGs at a given site that were methylated, were calculated for every sample at each CpG site. Observations with detection *P *>* *.05 were set to missing, and any CpG site with missing data was omitted from the analysis. The R programme, included in the SVA package, was used to adjust for the specific chip. A parametric empirical Bayes framework, that is a particularly robust method for dealing with small samples sizes, was utilized to adjust data for batch effects. For each site and each individual, β values were calculated by the following formula: β = signal_Meth_/(signal_meth_ + signal_Unmeth_).

### 2.4 Overall survival analysis

MethSurv performed univariable and multivariable survival analysis based on patient methylation levels for any CpG site (probe) using Cox proportional hazards models. In the univariable analysis, survival analysis was performed with probe methylation levels as explanatory variable and survival time as the response variable. In multivariable analysis, in addition to methylation status, clinical covariates such as age, sex and stage can be used. To assess differences in survival, methylation levels of patients were dichotomized into higher (the methylation β value higher than the cut‐off point) and lower groups (the methylation β value lower than the cut‐off point). Hazard ratio (HR) with 95% CI is derived from Cox fitting. The differences in survival between lower and higher methylated groups of patients were visualized by Kaplan‐Meier survival curve.

### 2.5 Cell culture

Thyroid cancer cell lines TPC‐1, FTC‐133 and normal thyroid follicular epithelial cell line of Nthy‐ori 3‐1 (BeNa Culture Collection, Beijing, China) were cultured in DMEM (Thermo Fisher Scientific, Waltham, MA, USA) supplemented with 10% FBS. Thyroid cancer cell line SW579 (BeNa Culture Collection) was cultured in Leibovitz's L‐15 medium (Thermo Fisher Scientific) containing 10% FBS.

### 2.6 RNA isolation and qRT‐PCR

Total RNA was isolated using Trizol reagent (Life Technologies), and then evaluated by gel electrophoresis and spectrophotometric analysis. First strand cDNA was generated through 5 mg of total RNA using the Superscript III‐reverse transcriptase kit (Invitrogen) based on the instructions, followed by dilution of the reaction mixture to 100 mL with water. 2.5 mL of diluted cDNA mixture was employed for PCR reaction. The cycling condition was 95°C 5 minutes, (95°C 30 seconds, 64°C 30 seconds, 72°C 40 seconds) 63 cycles, (95°C 30 seconds, 61°C 30 seconds, 72°C 40 seconds) 63 cycles, (95°C 30 seconds, 58°C 30 seconds, 72°C 40 seconds) 63 cycles, (95°C 30 seconds, 55°C 30 seconds, 72°C 40 seconds) 63 cycles, 72°C 7 minutes. β‐actin was served as an internal control. The primers sequence was provided in Table 1.

**Table 1:** qRT**‐**PCR primer sequence

Gene	Primer sequence(5′‐3′)
HORMAD2 (F)	5′ ‐GCAGCAGTACA AGCTTTGAAAGTGG‐3′
HORMAD2 (R)	5′ ‐TTGGTAATC ATGTGGGGTCACTGCA‐3′
β‐actin (F)	5′‐GGACTTCGAGCAAGAGATGG‐3′
β‐actin (R)	5′‐ AGCACTGTGTTGGCGTACAG‐3′

F, forward; R, reverse.

### 2.7 Methylation‐specific PCR (MSP)

Bisulfite‐treated DNA (50 ng) was mingled with AmpliTaq Gold polymerase (Applied Biosystems), mgCl2, and deoxynucleotide triphosphates for amplified reaction of MSP. The PCR amplification was carried out for 40 cycles at the annealing temperature of 60°C for 30 seconds. The final products were subjected to electrophoresis in a 2% agarose gel, and then recorded using a Molecular Imager (Bio‐Rad, Hercules, CA, USA). The methylation‐specific primers were listed in Table 2.

**Table 2:** The methylation‐specific PCR primers sequence

Gene	Primer sequence(5′‐3′)
HORMAD2 (MF)	5′‐ TTTGGGTTTAAGCGATTTTC‐3′
HORMAD2 (MR)	5′‐ CGCGTCACTTAAAATCAAAA‐3′
HORMAD2 (UF)	5′‐ TTTTTTGGGTTTAAGTGATTTTT‐3′
HORMAD2 (UR)	5′‐ AAACACATCACTTAAAATCAAAA‐3′
β‐actin (F)	5′‐ GGACTTCGAGCAAGAGATGG‐3′
β‐actin (R)	5′‐ AGCACTGTGTTGGCGTACAG‐3′

MF, methylation forward; MR, methylation reverse; UF, unmethylated forward; UR, unmethylated reverse; F, forward; R, reverse.

### 2.8 5‐aza‐2′‐deoxycytidine treatment

Thyroid cancer cell lines were treated with 5‐aza‐2′‐deoxycytidine (Sigma) to demethylate *HORMAD2* gene. For demethylation, the cells were cultured in the growth medium, to which 5‐aza‐2′‐deoxycytidine was added at a concentration of 2 mmol/L for 3 days before extraction of DNA using the Puregene DNA isolation kit (Gentra Systems Inc., Munich, Germany).

### 2.9 Western blot

A total of 20 μg proteins were added in 10% sodium dodecyl sulfate‐polyacrylamide gel, purified by centrifugalization, separated by electrophoresis and then shifted onto nitrocellulose membranes. The membranes were sealed with 5% skim milk for 1 hour and incubated with antibodies: rabbit anti‐*HORMAD2* (ab89961, Abcam, Cambridge, UK, dilution: 1:5000) overnight at 4°C and then with HRP‐conjugated mouse anti‐rabbit secondary antibody (1:2500) for 1 hour at 37°C. After washing with Tris‐Buffered Saline Tween‐20 (TBST) and Tris‐Buffered Saline (TBS), the protein bands were visualized via enhanced chemiluminescence (ECL) kit (Bio‐Rad). β‐actin was used as an internal control.

### 2.10 MTT assay

TPC‐1 and FTC‐133 cells treated or untreated with 5‐Aza were placed into 96‐well plates (3 × 10^3^ cells/well). The cell viability at 0, 24, 48 and 72 hours was determined through the MTT assay (#CGD1‐1KT, Sigma‐Aldrich, St. Louis, MO, USA). Absorbance was recorded on a microplate reader (Bio‐Rad) at a wavelength of 490 nm.

### 2.11 Flow cytometry analysis

The cell cycle and apoptosis were detected by flow cytometry. For apoptosis, both adherent and non‐adherent cells were collected and rinsed using pre‐cooled PBS twice and then resuspended in 200 μL binding buffer (10 mmol/L HEPES (pH7.4), 140 mmol/L NaCl, 1 mmol/L MgCl_2_, 5 mmol/L KCl, and 2.5 mmol/L CaCl_2_). FITC‐conjugated Annexin V was supplemented to a final concentration of 0.5 μg/mL and incubated for 20 minutes at 37°C in the dark, and 1 μg/mL PI was incorporated. Samples were immediately analysed by fluorescence‐activated cell sorting. Cells (1 × 10^4^) were recorded for apoptosis analysis. In terms of cell cycle, TPC‐1 cells were first digested and washed twice using PBS, fixed with ice‐cold 70% ethanol and then maintained at 4°C overnight. Subsequently, the cells were stained with PI (Thermo Fisher Scientific), and analysed by Cell Quest software (Becton Dickinson, Heidelberg, Germany). Having been washed twice with PBS, the stably transfected cells we collected were stained again with Annexin V‐FITC/PI in the dark. Likewise, the cell apoptosis was evaluated using Cell Quest software.

### 2.12 Transwell assay

The cell mobility and invasiveness were analysed through transwell assay. For cell migration, TPC‐1 and FTC‐133 cells were added to the upper chamber (8.0 mm pore size) of Transwell apparatus (Corning, NY, USA) at a density of 4 × 10^4^ cells (100 μL) each chamber and incubated for 13 hours, and then the cells that remained in the top chamber were removed with cotton swabs. For cell invasion, the upper chamber was covered with Matrigel (BD Biosciences). TPC‐1 and FTC‐133 cells were placed in the upper chamber at a density of 6 × 10^4^ cells (100 μL) each chamber. Following incubation for 36 hours, the cells that remained in the top chamber were scraped with cotton swabs, while cells penetrating into the lower membrane surface were fixed in 4% paraformaldehyde, stained with 0.2% crystal violet, and counted in high powered fields (10^6^) with light microscope.

### 2.13 Wound healing assay

TPC‐1 and FTC‐133 cells were seeded onto 6‐well plates, and linear scratch wounds in the confluent monolayers were created using a pipette tip. Inhibition of cell propagation was observed in medium containing FBS. Wound images at 0, 24 and 36 hours were obtained via a digital camera mounted on light microscope. The wound healing results were analysed using Image J software.

### 2.14 Tumour formation assay in vivo

Ten BALB/c nude mice (4‐5 weeks old, 18‐20 g, male) were purchased from Shanghai Experimental Animal Center (Shanghai, China) and randomly divided into 2 groups and labelled. The whole experimental procedure was authorized by Animal Ethics Committee in the First Hospital of Jilin University. 10^7^ TPC‐1 cells treated or untreated with 5‐Aza were injected subcutaneously into the left dorsal flank of nude mice, respectively. The length and width of tumour mass were measured every 3 days via a microcaliper. The tumour volume was calculated based on the equation of volume = (length × width^2^)/2. The mice were killed and the tumours were not taken out until the tumour length reached 1.5 cm. All tumour tissues were weighed before fixation in 4% paraformaldehyde and embedded in paraffin, and then prepared for subsequent immunohistochemical analysis.

### 2.15 Statistical analysis

The experimental data were analysed by Graphpad 6.0 (Version 6, CA, USA). Results were displayed in form of mean ± standard deviation. Comparison between the two sets of data was performed using Student's *t* test, while the differences between the three or more data were assessed by One‐way ANOVA. All experiments were repeated thrice. *P *<* *.05 signified a statistical significance.

## 3 RESULTS

### 3.1 Genome‐wide methylation data for thyroid cancer tissues

**Figure 1:**
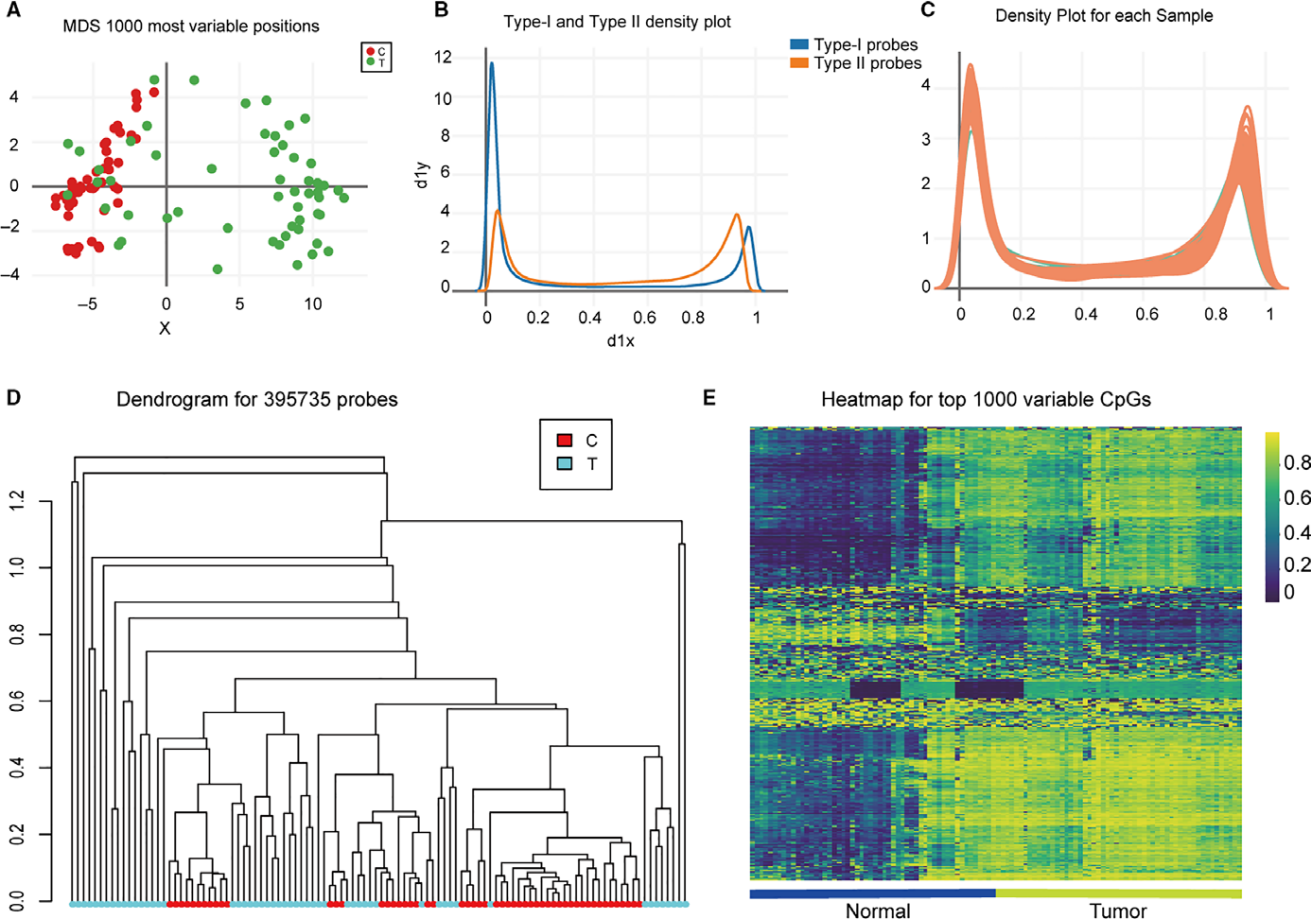
Genome‐wide methylation data was from TCGA for 54 available THCA tumour tissues and adjacent normal tissues. A, Multi‐dimensional scaling (MDS) plot showing differential clustering of control vs. thyroid cancer tissues. B, Density of methylated DNA intensity by probe type. Type‐I and Type‐II assays displayed different β‐value distributions. C, Methylation level distribution for each sample. *X*‐axis represents methylation level as mean β‐values. *Y*‐axis represents relative density (0 indicating unmethylated sites, 1 indicating fully methylated sites). D, Unsupervised hierarchical clustering analysis based on DNA methylation for 395 735 probes in control and thyroid cancer tissues. E, Heatmap of top 1000 differentially methylated imprinted CpG sites. The heatmap of hierarchical clustering analysis represents CpG sites methylation levels from completely methylated (dark blue) to unmethylated (green)

Multi‐dimensional scaling most 1000 variable positions showed that tumour and normal tissues had significantly different methylation level (Figure 1A). The density plot used in methylation analysis illustrated conspicuous difference between type I probes (blue line); type II probes (orange line) (Figure 1B). Each sample analysed displayed significant difference of methylation level between normal and tumour groups (Figure 1C). Unsupervised hierarchical clustering analysis was based on DNA methylation for 395 735 probes in control and tumour tissues (Figure 1D). The heatmap of hierarchical clustering analysis represented CpG sites methylation levels from completely methylated (dark blue) to unmethylated (green) (Figure 1E). These results indicated that the sample data we used exerted differential methylation level from two end‐points.

### 3.2 The methylation level of top 1000 differentially methylated CpG sites in imprinted genes

**Figure 2:**
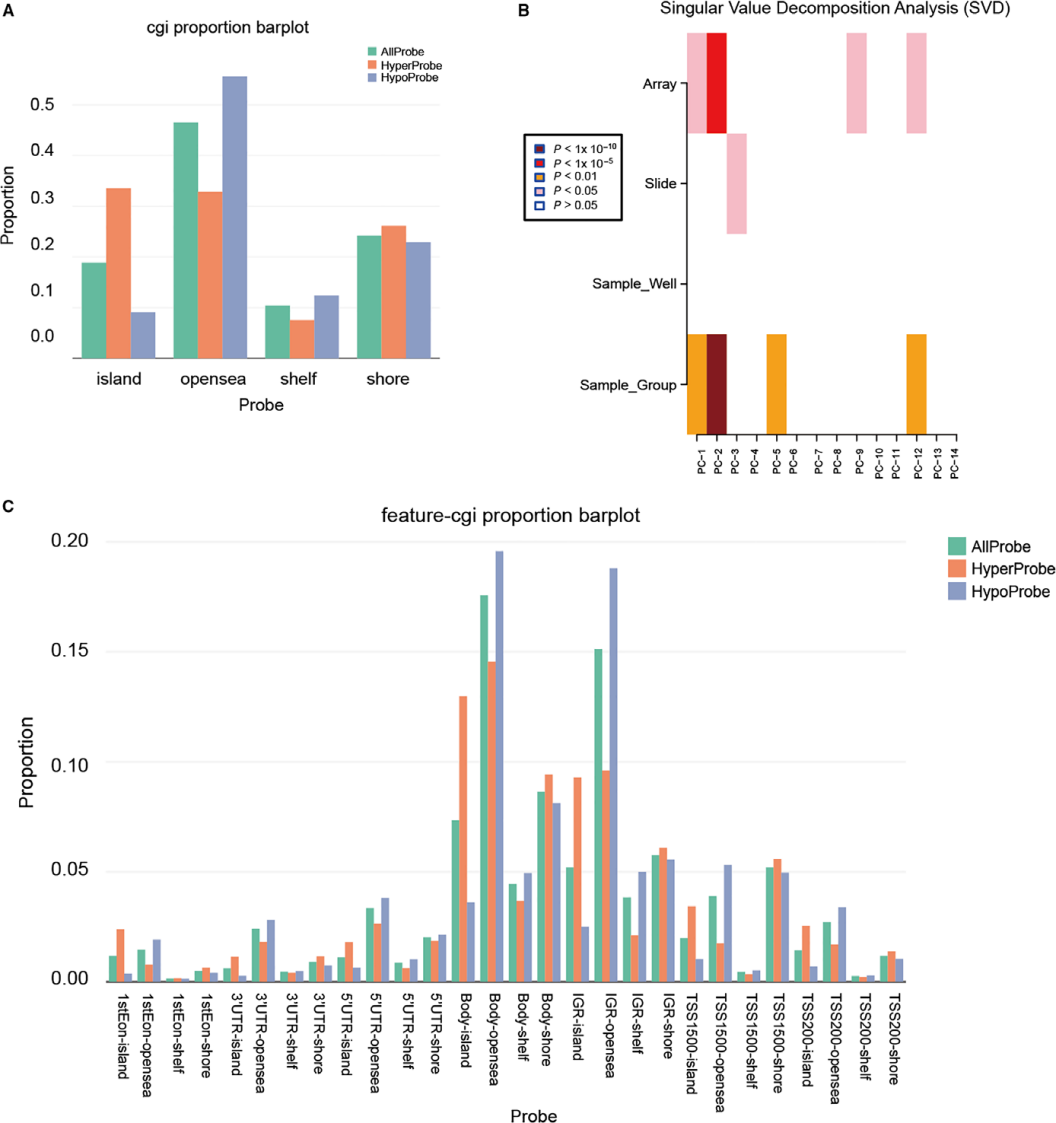
Distribution of top 1000 differentially methylated imprinted CpG sites. A, Distribution of top 1000 differentially methylated imprinted CpG sites according to CpG islands (shores, shelves, islands and open sea). B, Singular value decomposition analysis of 108 differentially methylated samples related with some important factors such as age, gender etc. C, Combining genetic and epigenetic annotation information revealed the distribution of top 1000 differentially methylated imprinted CpG sites

Probes were implemented within the Bioconductor package ChAMP and relied on a series of objects created using this package. CpG sites in the probes showed hypermethylation in island, while the methylation level decreased in open sea, shelf and shore (Figure 2A). SVD analysis showed if the difference between control and tumour groups is because of the samples themselves or other reasons (Figure 2B). The methylation of feature‐cgi proportion manifested the significant hypermethylation in 1stEon‐island and body‐island, whereas 1stEon‐opensea, body‐opensea, IGR‐opensea and IGR‐shelf presented a hypomethylation level (Figure 2C). These results illustrated the differential methylation level in all CpGs, where hypermethylation emerged in island region while the hypomethylation level appeared in open sea and shelf.

### 3.3 *HORMAD2* was hypermethylated in thyroid cancer tissues

**Figure 3:**
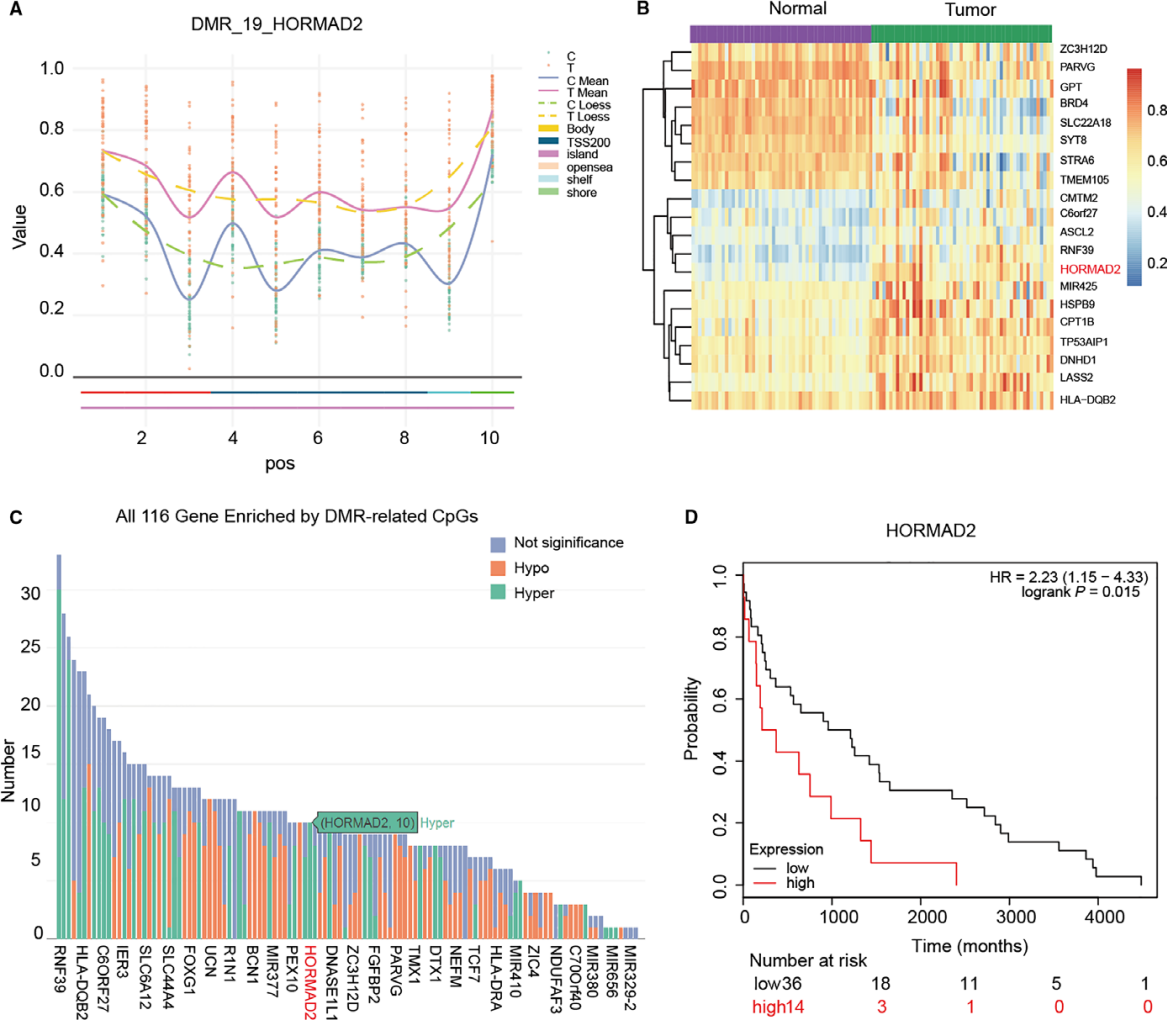
*HORMAD2* was hypermethylated in THCA tissue. A, The differently methylated region DMR_19 enriched with *HORMAD2* was significantly hypermethylated in tumour tissue. B, The heatmap of hierarchical clustering analysis represents top 20 differentially methylated genes. Epigenetic distances (Euclidean Distance) were calculated by microarray Array. C, All 116 genes enriched by differently methylated region‐related CpGs and *HORMAD2* was hypermethylated. D, Prognostic value of the methylation status of *HORMAD2*. Patients with a higher expression of *HORMAD2* had a lower survival rate

**Figure 4:**
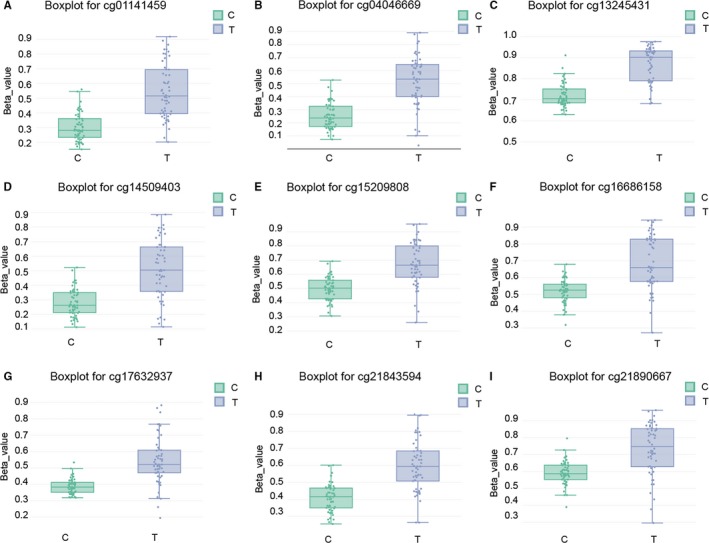
Nine of the most remarkable methylation level of CpG sites for *HORMAD2*. Boxplots showed higher methylation level of cg01141459 (A), cg04046669 (B), cg13245431 (C), cg14509403 (D), cg15209808 (E) cg16686158 (F), cg17632937 (G), cg2184594 (H), cg21890667 (I) in the thyroid cancer tissue

The differentially methylated region DMR_19 enriched with *HORMAD2* was significantly hypermethylated in thyroid cancer tissues (Figure 3A) and the heatmap displayed top 20 different methylation genes enriched in THCA tissues, in which *HORMAD2* was hypermethylated (Figure 3B). ALL 116 genes enriched by DMR‐related CpGs had different methylation level, which indicated that *HORMAD2* was significantly hypermethylated in THCA tissues (Figure 3C). Survival prognosis curve revealed that high methylation level of *HORMAD2* was correlated with poor prognosis in THCA patients (Figure 3D). Based on the methylation levels, RFS of 54 THCA patients was a continuous variable, which could be considered as prognostic factors. RFS referred to the time between initial diagnosis and recurrence or death, with follow‐up censored at last contact if no event had occurred. The methylation level of top 9 CpGs located in *HORMAD2* were shown in Boxplot for cg01141459, cg04046669, cg13245431, cg14509403, cg15209808, cg16686158, cg17632937, cg2184594 and cg21890667, respectively (Figure 4A‐I). The above results illustrated the methylation level in THCA group was remarkably higher compared with control group.

### 3.4 The expression of *HORMAD2* was down‐regulated by hypermethylation in thyroid cancer

**Figure 5:**
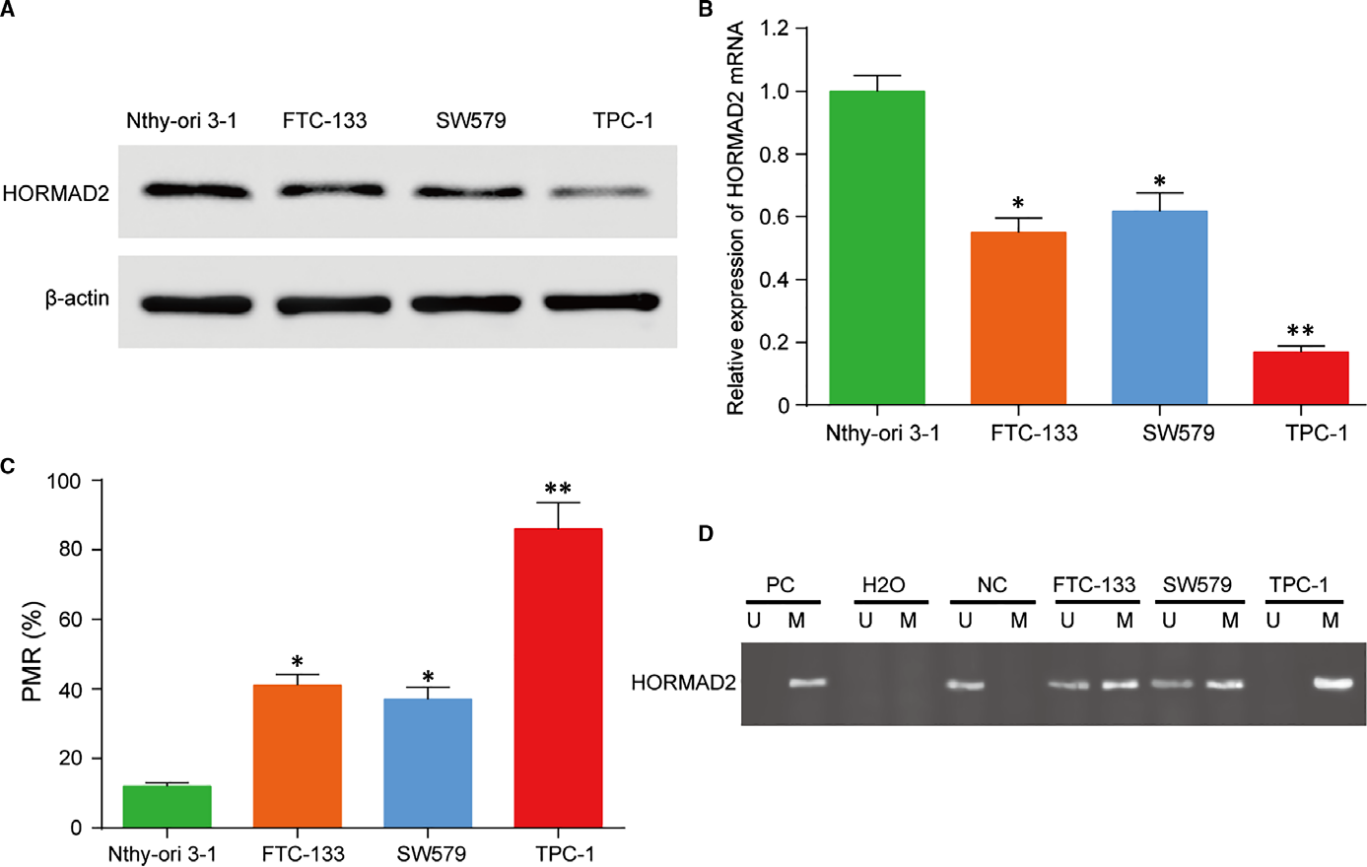
The methylation level and gene expression of *HORMAD2* in tumour and normal cells. A and B, The *HORMAD2* expression in thyroid cancer cell lines FTC‐133, SW579, TPC‐1 and thyroid follicular epithelial cell line of Nthy‐ori 3‐1. *HORMAD2* was highly expressed in Nthy‐ori 3‐1 cells and its expression decreased in FTC‐133 and SW579 cells but significant lowly expressed in TPC‐1 cells. C and D, MSP results showed *HORMAD2* methylation status. Complete methylation was found in TPC‐1 cells, and partial methylation was found in FTC‐133 and SW579 cells, little methylation was found in nthy‐ori 3‐1 cells. PC (invitro methylation DNA) served as methylation positive control. NC (normal lymphocyte DNA) served as unmethylation negative control). M, methylated alleles; U, unmethylated alleles; **P <* *.01 compared with Nthy‐ori 3‐1 cells

The expression of *HORMAD2* was detected by qRT‐PCR and Western blot in human thyroid cancer cells and normal thyroid follicular epithelial cells. The expression of *HORMAD2* was high in normal thyroid follicular epithelial cell lines of Nthy‐ori 3‐1 cells but decreased markedly in thyroid cancer cells of FTC‐133, SW579 and TPC‐1 cells, especially in TPC‐1cells (Figure 5A,B, *P* < .01). The methylation level of *HORMAD2* was examined by methylation‐specific PCR (MSP). A complete methylation was detected in TPC‐1 cells, and partial methylation was found in FTC‐133 and SW579 cells, little methylation was found in Nthy‐ori 3‐1 cells (Figure 5C,D, *P* < .01). These results indicated that reduced expression of *HORMAD2* is correlated with hypermethylation in thyroid cell lines. These results demonstrated that *HORMAD2* expression is regulated by methylation in human thyroid cancer cells and we select TPC‐1 cells for subsequent research.

### 3.5 *HORMAD2* suppressed cell migration, invasion and proliferation in thyroid cancer cells

**Figure 6:**
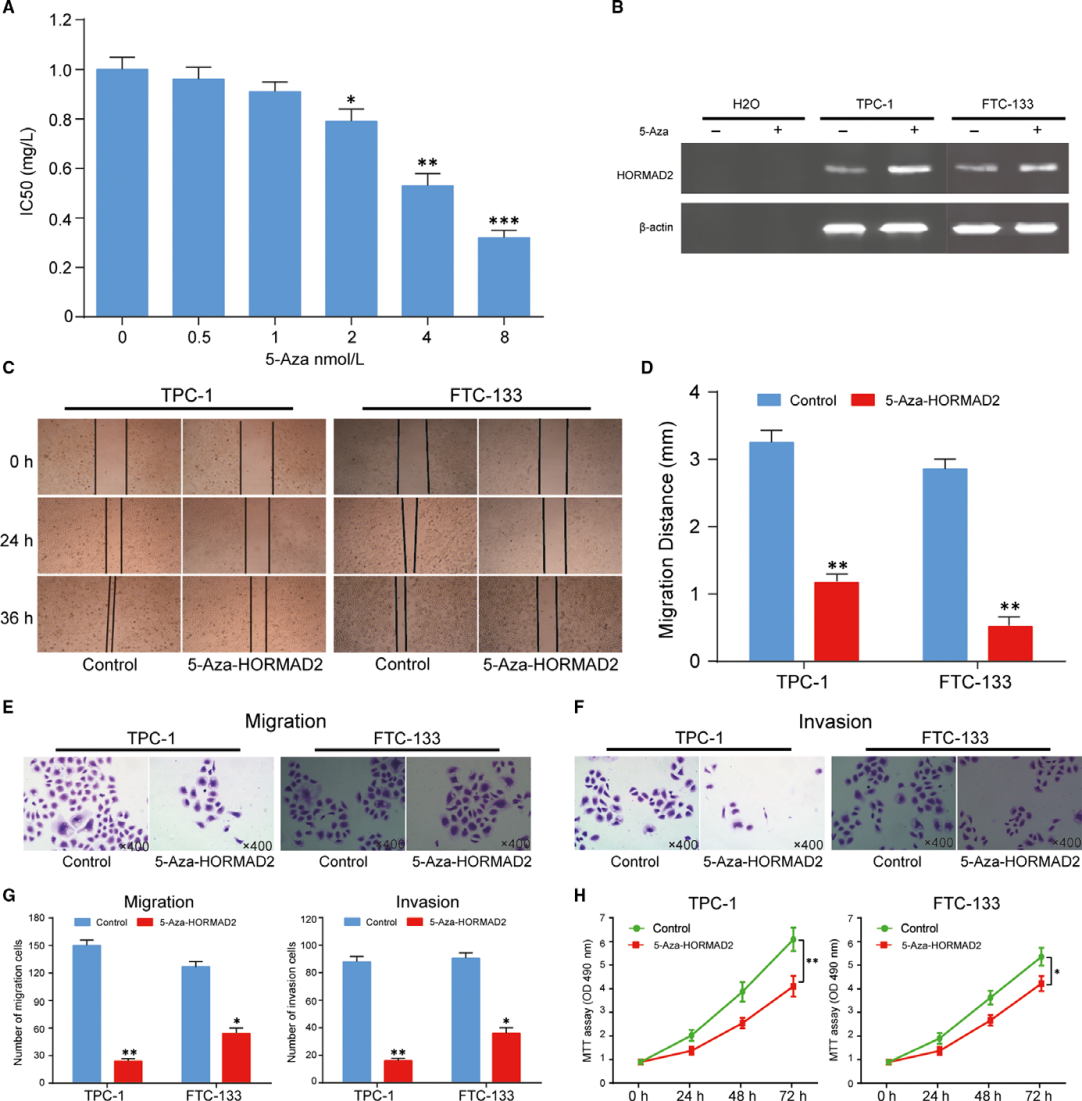
The migration, invasion and proliferation were suppressed by *HORMAD2* in thyroid cancer cells. A, Minimum effective dose of 5‐Aza determined by MTT assay and 2 nmol/L was used in our experiment. *P <* *.05, **P <* *.01, ***P <* *.001 represented compare with 0 nmol/L group. B, The effect of 5‐Aza on *HORMAD2* expression in TPC‐1 and FTC‐133 cells was detected by Western blot, indicating demethylation of *HORMAD2* was highly expressed in TPC‐1 and FTC‐133 cells. C and D, Cell migration was suppressed by *HORMAD2* in TPC‐1 and FTC‐133 cells under the wound healing detection. Blue column represents the wound healing result of normal TPC‐1 and FTC‐133 cells and the red column represents the wound healing result of *HORMAD2* re‐expressed group in TPC‐1 and FTC‐133 cells for 36 h, **P <* *.01 compared with control group. E‐G, Cell migration and invasion were suppressed by *HORMAD2* in TPC‐1 and FTC‐133 cells, **P <* *.01 compared with control group in TPC‐1 cells. *P <* *.05 compared with control group in FTC‐133 cells. H, The effect of *HORMAD2* on cell viability was measured by the MTT assay for 72 h in TPC‐1 and FTC‐133 cells, **P <* *.01 compared with control group in TPC‐1 cells. *P <* *.05 compared with control group in FTC‐133 cells

We next assessed the effects of 5‐Aza on *HORMAD2* expression and the concentration of 5‐Aza in experimental analysis was used at the minimum effective dose (2 nmol/L) in Figure 6A. The effect of 5‐Aza on *HORMAD2* expression was shown in Figure 6B. *HORMAD2* was up‐regulated significantly after treatment with 5‐Aza in TPC‐1 cells and the trend was not very noticeable in FTC‐133 cells. To examine the influence of *HORMAD2* on cell mobility, invasion and proliferation, transwell assay, wound healing assays and MTT assay were employed. As shown in Figure 6C,D, the migration distance was sharply suppressed after re‐expression of *HORMAD2* in TPC‐1 cells compared with untreated TPC‐1 cells (control group) (*P *<* *.01). In FTC‐133 cells, the migration distance was also suppressed after re‐expression of *HORMAD2*, but less inhibited than TPC‐1 cells. Under the Transwell assay, the number of migratory cells and invasive cells were reduced significantly after re‐expression of *HORMAD2* in TPC‐1 cells (Figure 6E‐G). While the number of migratory cells and invasive cells was inhibited after re‐expression of *HORMAD2* in FTC‐133 cells and the degree of inhibition was lower than TPC‐1 cells. These results suggested that *HORMAD2* restrained TCHA cell mobility and invasiveness. The MTT assay result showed cell viability was reduced remarkably after restoration of *HORMAD2* in TPC‐1 cells compared with untreated TPC‐1 cells. Besides, the results of MTT assay in FTC‐133 cells were also inhibited (Figure 6H).

### 3.6 *HORMAD2* promoted thyroid cell apoptosis and suppressed cells mitosis

**Figure 7:**
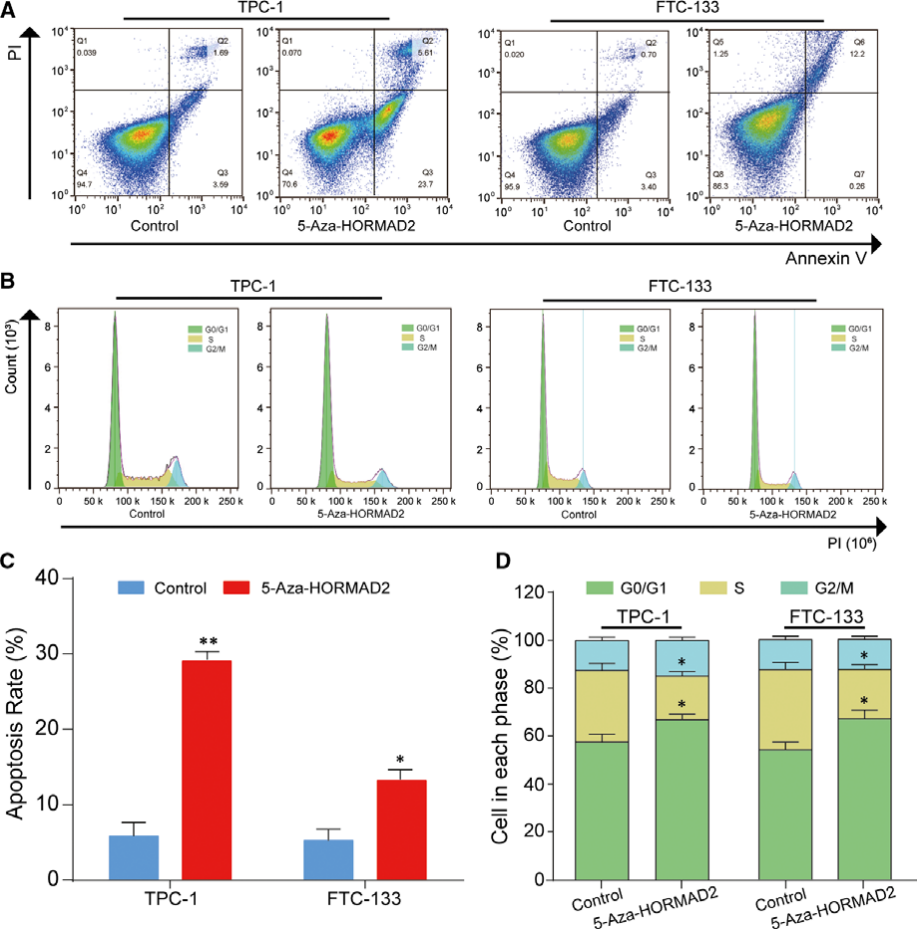
*HORMAD2* promote tumour cells apoptosis and inhibit tumour growth. A and C, Apoptosis determined by flow cytometry. Cell apoptosis was increased in TPC‐1 and FTC‐133 cells treated with 5‐Aza, **P <* *.01 compared with control group in TPC‐1 cells. *P <* *.05 compared with control group in FTC‐133 cells. B and D, The effect of *HORMAD2* on cell cycle was determined by flow cytometry. 5‐Aza‐*HORMAD2* group had longer G0/G1 term and shorter S term than control group in TPC‐1 and FTC‐133 cells, **P <* *.01 compared with control group

The impacts of *HORMAD2* on cell apoptosis and cell cycle were detected by flow cytometry. The results showed obvious increase of apoptotic after treatment with 5‐Aza in TPC‐1cells and FTC‐133 cells compared with untreated TPC‐1 cells and FTC‐133 cells (Figure 7A,C). At the meanwhile, *HORMAD2* treated with 5‐Aza could significantly inhibit mitosis in TPC‐1 cells by prolonging G0/G1 phase and shortening S phase compared with untreated TPC‐1 cells (Figure 7B,D; *P* < .01). The cell cycle in FTC‐133 cells was also detected and the results showed that more cells were arrested in G0/G1 phase. This outcome implied that demethylation of *HORMAD2* can induce more apoptotic thyroid cancer cells as well as inhibit thyroid cancer cells mitosis.

### 3.7 *HORMAD2* suppressed thyroid tumour growth in vivo

**Figure 8:**
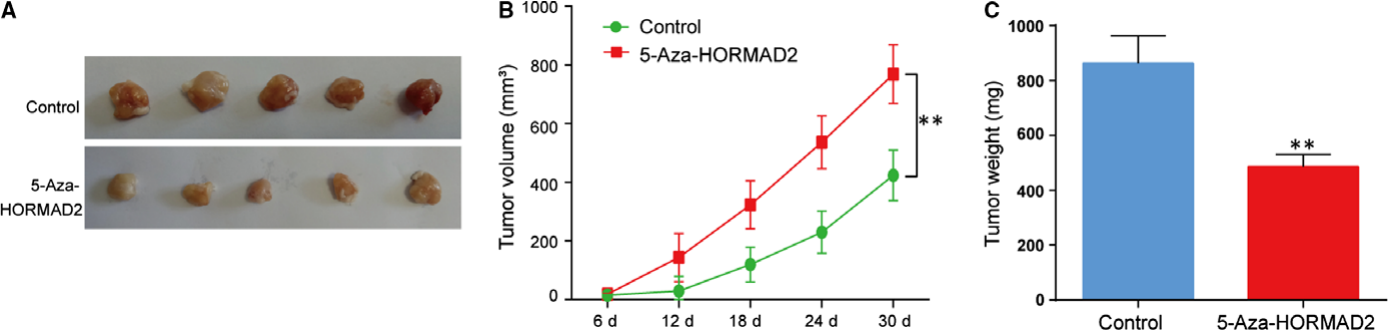
*HORMAD2* suppresses cancer cell xenograft growth in vivo. A, Representative results of xenograft tumours in mice in 5‐Aza‐*HORMAD2* group and control group. B, The tumour growth curve and C, tumour weight of 5‐Aza‐*HORMAD2* group and control group showed *HORMAD2* could suppress tumour growth, **P <* *.01 compared with control group

The xenograft mouse model was established (Figure 8A) and the tumour volume was significantly smaller in *HORMAD2* treated with 5‐Aza in TPC‐1 cell line xenografts than normal TPC‐1 cell line xenografts (Figure 8B, *P* < .01). The tumour weight decreased largely after *HORMAD2* treated with 5‐Aza in xenograft mouse model (Figure 8C, *P* < .01). The result illustrated *HORMAD2* can suppress tumour growth to a large degree.

## 4 DISCUSSION

In this study, we delved into the connections between epigenetic regulation and THCA pathogenesis via sequencing the whole genome DNA methylation and gene expression of the tumour tissues and normal thyroid tissues from THCA patients. Our research confirmed the methylation level of *HORMAD2* and its critical role in THCA progression for the first time and provided a deeper insight into the pathogenesis of THCA.

Thyroid carcinomas are classified into the following four representative histological types: papillary, follicular, medullary and anaplastic carcinomas.^17^ TPC‐1 thyroid cancer cell line was derived from papillary thyroid cancer, while FTC‐133 thyroid cancer cell line was derived from follicular thyroid cancer.^18^ Thyroid papillary carcinoma was the most common and the lowest degree of malignancy. It accounts for 85% of thyroid cancer and can develop at any age. Okada et al^17^ found that the expression of EpCAM in two anaplastic thyroid cancer cell lines (FRO and ACT‐1) were remarkably higher than those in the TPC‐1 and FTC‐133 cells. D‘Agostino et al clearly indicated that TSHR and NIS transcript levels in FTC‐133 cells are subjected to different forms of epigenetic control.^19^ We focused on detecting the methylation level of *HORMAD2* and its impact on different subtype thyroid carcinoma (THCA) cells (TPC‐1 and FTC‐133) development.

DNA methylation is known to result in the transcriptional inactivation of tumour suppressors in the early stage of cancer. Along with these genetic factors, epigenetic regulation plays a crucial role in the initiation and progression of various types of cancers.^20^ Epigenetic alterations, such as aberrant promoter methylation, can yield powerful biomarkers for early detection of diseases.^21^, ^22^, ^23^ A previous research of Belinsky et al^24^ displayed that aberrant methylation of the *p16* might be involved in hepatocellular cancer, which might be a novel biomarker for early detection. Takane et al^25^ found the aberrant promoter methylation of *PPP1R3C* and *EFHD1* in plasma of colorectal cancer (CRC) patients, and verified that both of them could be potential detection markers for CRC. Kikuchi et al^26^ identified multiple genes (*HIST1H3J, POU4F2, SHOX2, PHKG2, TLX3* and *HOXA7*) with frequent epigenetic hypermethylation in papillary thyroid cancer, which might function as potential biomarkers. Herein, we conducted a genome‐wide DNA methylation analysis so as to identify epigenetically regulated molecular players in THCA. We mainly focused on the epigenetic regulation and functions of *HORMAD2*, and demonstrated that *HORMAD2* expression could be modulated by promoter region methylation. These data suggested that *HORMAD2* was implicated in THCA initiation and progression. Meanwhile, we hypothesized that aberrant methylation of the tumour suppressor or gene promoter, containing *HORMAD2* promoter, might further lead to an abnormal alteration of genome methylation in the late stages of THCA.

It is recognized that HORMA‐domain containing proteins (HORMAD) plays a crucial role in the regulation of cell cycle and in mitosis and meiosis.^27^, ^28^, ^29^ HORMADs (HORMAD1 and HORMAD2) share homology with the meiosis‐specific HORMA‐domain proteins, which are conserved in various organisms.^16^ *HORMAD1* has been shown to be aberrantly expressed in multiple diseases and was considered to be a potentially important oncogene in some former studies.^30^ *HORMAD2*, as another HORMA‐domain encoding gene, has been also reported in a wide variety of cancers, primarily by means of epigenetic mechanisms.^31^ For instance, Liu et al^32^ confirmed that *HORMAD2* was ectopically expressed in hepatocellular cancer (HCC) tissues and it might be a candidate biomarker for risk of HCC. Herein, we mainly studied on methylation status of *HORMAD2* promoter, and verified for the first time that *HORMAD2* was aberrantly methylated in THCA in tissues and cells, which might lead to cancer progression and tumorigenesis.

Additionally, the present study also examined the impact of 5‐aza‐2′‐deoxycytidine (5‐Aza) on the methylation of *HORMAD2* and biological functions of TPC‐1 cells in vivo and in vitro. 5‐Aza, a DNA methyltransferase (DNMT) inhibitor, leads to DNMT inactivation through DNMT covalent bonding with thiol on cysteine residues, causing reactivation genes silenced by promoter methylation.^33^ Accumulative researches reported the significance of 5‐Aza in the inhibition of hypermethylation and restoration of gene expression. Zhang et al^34^ uncovered that reduction of *DACT2* expression was associated with promoter hypermethylation and restoration of *DACT2* expression could be induced by 5‐Aza in human hepatocellular carcinoma. Sassa et al^35^ revealed that pharmacological inhibition of methylation in TPC‐1 cells by 5‐Aza resulted in increased expression of *CITED1*, thereby influencing papillary thyroid cancer progression. Wu et al^36^ verified that restoration of *HIC1* expression via 5‐Aza treatment reduced *SIRT1* expression and cell propagation, and led to senescence, cell cycle arrest and apoptosis in thyroid papillary carcinoma. Likewise, our results were consistent with the above previous findings that 5‐Aza could significantly suppressed *HORMAD2* hypermethylation and impeded THCA progression through increasing its expression.

Nevertheless, there were still some deficiencies worthy to be mentioned in this study. First, our research roughly revealed the *HORMAD2* methylation‐mediated epigenetic regulation mechanism in THCA, instead of in some certain subtype of THCA. Thus the generality of the results in different thyroid cancer subtypes remained to be further verified. Second, MSP technique we used for detection of methylation level could be more qualitative than quantitative and less useful for the assessment of genome‐wide methylation changes.

In summary, *HORMAD2* was aberrantly methylated in THCA and its promoter hypermethylation could regulate *HORMAD2* expression. The addition of 5‐Aza significantly suppressed the methylation level of *HORMAD2*, and hence increased the mRNA expression. Hypermethylation of *HORMAD2* could induce THCA progression, while hypomethylation of *HORMAD2* retarded cell growth and mobility and facilitated apoptosis. Our study demonstrated for the first time that *HORMAD2* might contribute to THCA progression through methylation‐mediated epigenetic regulation of gene expression, and discovered a novel molecular diagnostic marker and therapeutic target for THCA.

## CONFLICT OF INTEREST

The authors confirm that there are no conflict of interests.

## References

[1] Li P , Yang W , Shen B , Li H , Yan J . Lentivirus‐mediated silencing of mphosph8 inhibits mtc proliferation and enhances apoptosis. Oncol Lett. 2016;11:4117‐4122. 27313751 10.3892/ol.2016.4545 PMC4888193

[2] Ferrari SM , Fallahi P , Politti U , et al. Molecular targeted therapies of aggressive thyroid cancer. Front Endocrinol. 2015;6:176. 10.3389/fendo.2015.00176 PMC4653714 26635725

[3] Verdelli C , Forno I , Vaira V , Corbetta S . Epigenetic alterations in human parathyroid tumors. Endocrine. 2015;49:324‐332. 25722013 10.1007/s12020-015-0555-4

[4] Nikiforov YE , Nikiforova MN . Molecular genetics and diagnosis of thyroid cancer. Nat Rev Endocrinol. 2011;7:569‐580. 21878896 10.1038/nrendo.2011.142

[5] Nikiforova MN , Nikiforov YE . Molecular genetics of thyroid cancer: implications for diagnosis, treatment and prognosis. Expert Rev Mol Diagn. 2008;8:83‐95. 18088233 10.1586/14737159.8.1.83

[6] Shin JJ , Milas M . Detection of disease recurrence in differentiated thyroid cancer. Minerva Chir. 2010;65:101‐116. 20212422

[7] Showalter TN , Siegel BA , Moley JF , Baranski TJ , Grigsby PW . Prognostic factors in patients with well‐differentiated thyroid cancer presenting with pulmonary metastasis. Cancer Biother Radiopharm. 2008;23:655‐659. 18976119 10.1089/cbr.2008.0501

[8] Zhu X , Li F , Yang B , Liang J , Qin H , Xu J . Effects of ultraviolet b exposure on DNA methylation in patients with systemic lupus erythematosus. Exp Ther Med. 2013;5:1219‐1225. 23596493 10.3892/etm.2013.960 PMC3628076

[9] Shen J , Wang S , Zhang YJ , et al. Exploring genome‐wide DNA methylation profiles altered in hepatocellular carcinoma using infinium humanmethylation 450 beadchips. Epigenetics. 2013;8:34‐43. 23208076 10.4161/epi.23062 PMC3549879

[10] Wehbe H , Henson R , Meng F , Mize‐Berge J , Patel T . Interleukin‐6 contributes to growth in cholangiocarcinoma cells by aberrant promoter methylation and gene expression. Can Res. 2006;66:10517‐10524. 10.1158/0008-5472.CAN-06-2130 17079474

[11] Watson RE , Curtin GM , Hellmann GM , Doolittle DJ , Goodman JI . Increased DNA methylation in the hoxa5 promoter region correlates with decreased expression of the gene during tumor promotion. Mol Carcinog. 2004;41:54‐66. 15352125 10.1002/mc.20043

[12] Lal G , Padmanabha L , Smith BJ , et al. Riz1 is epigenetically inactivated by promoter hypermethylation in thyroid carcinoma. Cancer. 2006;107:2752‐2759. 17103461 10.1002/cncr.22325

[13] Quackenbush J . Microarray analysis and tumor classification. N Engl J Med. 2006;354:2463‐2472. 16760446 10.1056/NEJMra042342

[14] Hou P , Liu D , Xing M . Genome‐wide alterations in gene methylation by the braf v600e mutation in papillary thyroid cancer cells. Endocr Relat Cancer. 2011;18:687‐697. 21937738 10.1530/ERC-11-0212 PMC3346957

[15] Nikolova DN , Zembutsu H , Sechanov T , et al. Genome‐wide gene expression profiles of thyroid carcinoma: identification of molecular targets for treatment of thyroid carcinoma. Oncol Rep. 2008;20:105‐121. 18575725

[16] Wojtasz L , Daniel K , Roig I , et al. Mouse hormad1 and hormad2, two conserved meiotic chromosomal proteins, are depleted from synapsed chromosome axes with the help of trip13 aaa‐atpase. PLoS Genet. 2009;5:e1000702. 19851446 10.1371/journal.pgen.1000702 PMC2758600

[17] Okada T , Nakamura T , Watanabe T , et al. Coexpression of epcam, cd44 variant isoforms and claudin‐7 in anaplastic thyroid carcinoma. PLoS ONE. 2014;9:e94487. 24727741 10.1371/journal.pone.0094487 PMC3984167

[18] Arcinas A , Yen TY , Kebebew E , Macher BA . Cell surface and secreted protein profiles of human thyroid cancer cell lines reveal distinct glycoprotein patterns. J Proteome Res. 2009;8:3958‐3968. 19530676 10.1021/pr900278c PMC2735218

[19] D'Agostino M , Sponziello M , Puppin C , et al. Different expression of tsh receptor and nis genes in thyroid cancer: role of epigenetics. J Mol Endocrinol. 2014;52:121‐131. 24353283 10.1530/JME-13-0160

[20] Kondo T , Asa SL , Ezzat S . Epigenetic dysregulation in thyroid neoplasia. Endocrinol Metab Clin North Am. 2008;37:389‐400, ix. 18502333 10.1016/j.ecl.2007.12.002

[21] Zhao Z , Herman JG , Brock MV , et al. Methylation of dact2 promotes papillary thyroid cancer metastasis by activating wnt signaling. PLoS ONE. 2014;9:e112336. 25375359 10.1371/journal.pone.0112336 PMC4223043

[22] Brown TC , Juhlin CC , Healy JM , Prasad ML , Korah R , Carling T . Frequent silencing of rassf1a via promoter methylation in follicular thyroid hyperplasia: a potential early epigenetic susceptibility event in thyroid carcinogenesis. JAMA Surg. 2014;149:1146‐1152. 25229773 10.1001/jamasurg.2014.1694

[23] Dumitrescu RG . Epigenetic markers of early tumor development. Methods Mol Biol. 2012;863:3‐14. 22359284 10.1007/978-1-61779-612-8_1

[24] Belinsky SA , Nikula KJ , Palmisano WA , et al. Aberrant methylation of p16(ink4a) is an early event in lung cancer and a potential biomarker for early diagnosis. Proc Natl Acad Sci USA. 1998;95:11891‐11896. 9751761 10.1073/pnas.95.20.11891 PMC21736

[25] Takane K , Midorikawa Y , Yagi K , et al. Aberrant promoter methylation of ppp1r3c and efhd1 in plasma of colorectal cancer patients. Cancer Med. 2014;3:1235‐1245. 24861485 10.1002/cam4.273 PMC4302673

[26] Kikuchi Y , Tsuji E , Yagi K , et al. Aberrantly methylated genes in human papillary thyroid cancer and their association with braf/ras mutation. Front Genet. 2013;4:271. 24367375 10.3389/fgene.2013.00271 PMC3851831

[27] Shin YH , Choi Y , Erdin SU , et al. Hormad1 mutation disrupts synaptonemal complex formation, recombination, and chromosome segregation in mammalian meiosis. PLoS Genet. 2010;6:e1001190. 21079677 10.1371/journal.pgen.1001190 PMC2973818

[28] Fukuda T , Daniel K , Wojtasz L , Toth A , Höög C . A novel mammalian horma domain‐containing protein, hormad1, preferentially associates with unsynapsed meiotic chromosomes. Exp Cell Res. 2010;316:158‐171. 19686734 10.1016/j.yexcr.2009.08.007

[29] Chen YT , Venditti CA , Theiler G , et al. Identification of ct46/hormad1, an immunogenic cancer/testis antigen encoding a putative meiosis‐related protein. Cancer Immun. 2005;5:9. 15999985

[30] Adelaide J , Finetti P , Bekhouche I , et al. Integrated profiling of basal and luminal breast cancers. Can Res. 2007;67:11565‐11575. 10.1158/0008-5472.CAN-07-2536 18089785

[31] Lawson C , Gieske M , Murdoch B , et al. Gene expression in the fetal mouse ovary is altered by exposure to low doses of bisphenol A. Biol Reprod. 2011;84:79‐86. 20739668 10.1095/biolreprod.110.084814 PMC3012563

[32] Liu M , Chen J , Hu L , et al. Hormad2/ct46.2, a novel cancer/testis gene, is ectopically expressed in lung cancer tissues. Mol Hum Reprod. 2012;18:599‐604. 22893617 10.1093/molehr/gas033

[33] Liu R , Zhang XH , Zhang K , et al. 5‐aza‐2′’‐deoxycytidine inhibits retinoblastoma cell by reactivating epigenetically silenced rassf1a gene. Int J Ophthalmol. 2014;7:51‐56. 24634863 10.3980/j.issn.2222-3959.2014.01.09 PMC3949458

[34] Zhang X , Yang Y , Liu X , et al. Epigenetic regulation of the wnt signaling inhibitor dact2 in human hepatocellular carcinoma. Epigenetics. 2013;8:373‐382. 23449122 10.4161/epi.24113 PMC3674046

[35] Sassa M , Hayashi Y , Watanabe R , et al. Aberrant promoter methylation in overexpression of cited1 in papillary thyroid cancer. Thyroid. 2011;21:511‐517. 21449767 10.1089/thy.2010.0295

[36] Wu W , Zhang L , Lin J , et al. Hypermethylation of the hic1 promoter and aberrant expression of hic1/sirt1 contribute to the development of thyroid papillary carcinoma. Oncotarget. 2016;7:84416‐84427. 27793057 10.18632/oncotarget.12936 PMC5356670

